# Herpes simplex virus 1 evades cellular antiviral response by inducing microRNA-24, which attenuates STING synthesis

**DOI:** 10.1371/journal.ppat.1009950

**Published:** 2021-09-30

**Authors:** Nikhil Sharma, Chenyao Wang, Patricia Kessler, Ganes C. Sen

**Affiliations:** Department of Inflammation and Immunity, Lerner Research Institute, Cleveland Clinic, Cleveland, Ohio, United States of America; Duke University Medical Center, UNITED STATES

## Abstract

STING is a nodal point for cellular innate immune response to microbial infections, autoimmunity and cancer; it triggers the synthesis of the antiviral proteins, type I interferons. Many DNA viruses, including Herpes Simplex Virus 1 (HSV1), trigger STING signaling causing inhibition of virus replication. Here, we report that HSV1 evades this antiviral immune response by inducing a cellular microRNA, miR-24, which binds to the 3’ untranslated region of STING mRNA and inhibits its translation. Expression of the gene encoding miR-24 is induced by the transcription factor AP1 and activated by MAP kinases in HSV1-infected cells. Introduction of exogenous miR-24 or prior activation of MAPKs, causes further enhancement of HSV1 replication in STING-expressing cells. Conversely, transfection of antimiR-24 inhibits virus replication in those cells. HSV1 infection of mice causes neuropathy and death; using two routes of infection, we demonstrated that intracranial injection of antimiR-24 alleviates both morbidity and mortality of the infected mice. Our studies reveal a new immune evasion strategy adopted by HSV1 through the regulation of STING and demonstrates that it can be exploited to enhance STING’s antiviral action.

## Author summary

The type I interferon system is the first line of cellular antiviral innate immune response. Virus infection is recognized by various pattern recognition receptors in the infected cell and it activates the interferon system to inhibit virus replication. However, viruses have evolved various mechanisms to evade the cellular immune response and enhance viral replication. Our study uncovers an immune evasion strategy used by the Herpes Simplex virus to circumvent the cGAS/STING signaling pathway which is the pivotal innate immune response to combat DNA virus replication. miR-24 induction by HSV1 targets STING and hence, dampens Type I Immune response against the virus. The induction of miR-24 is regulated by virus induced MAPK activation, which are also required during early lytic cycles of HSV1 replication and is indispensable for HSV1 reactivation from latency in neurons; depicting a new direct co-relation between MAPK activation and HSV1 replication orchestrated through cellular miR-24. Silencing of miR-24 in mice brain curtails viral replication and disease severity. Overall, these results indicate possible therapeutic use of stable antimiR-24 against HSV1 and other diseases that are alleviated by STING.

## Introduction

Microbial infections and other external stresses are recognized by pattern recognition receptors (PRRs), which trigger defense responses to maintain cellular homeostasis [1]. Pattern recognition receptors, such as RIG-I like receptors (RLR), Toll-like receptors (TLR) and Nod like receptors (NLR) recognize various pathogen associated molecular patterns (PAMP) and trigger the first line of innate immune defense against the invading pathogens [2,3]. DNA viruses are recognized by PRRs, cGAS, IFI16 and DDX41, in the infected cell, and thereby orchestrate a cascade of signaling to elicit antiviral Type I IFN response [4,5]. STING is an endoplasmic reticulum protein, which is required for signaling by these PRRs [6]. Cytosolic viral DNA, sensed by cGAS, leads to the synthesis of cyclic guanosine adenosine monophosphate (cGAMP), which activates STING [7]. Cytoplasmic leakage of nuclear or mitochondrial DNA in an infected cell or a tumor cell can also be recognized by cGAS and STING [8,9]. In addition, many intracellular bacteria synthesize different cyclic dinucleotides, which can activate STING, without any help from cGAS [10]. Therefore, STING is a pivotal protein required for eliciting cellular defense response against a variety of stresses.

Herpes simplex virus 1 (HSV1), a DNA virus, has infected majority of the human population and undergoes latency in neuronal ganglions [11,12]. HSV1 infects the mucosal surfaces and penetrates into neurons to establish latency and it reactivates frequently causing blisters, neuralgia and encephalitis [13]. Infection of cells in culture by HSV1 triggers STING signaling and IFN synthesis [14]. IFN produced by the infected cells protects neighboring cells from virus infection, thus limiting virus replication in the population [15]. Consequently, the absence of STING or any protein necessary for signaling by it, promotes HSV1 replication [16,17]. As expected, STING -/- mice are more susceptible to HSV1 infection and virus induced encephalitis [18]. Viruses have evolved various strategies to evade cellular immune responses [19,20]. To evade the antiviral action of STING, HSV1 engages multiple strategies [21,22]. Here, we report a new strategy, in which the virus induces the synthesis of a cellular microRNA, which inhibits STING synthesis.

MicroRNAs are small regulatory RNAs which bind to the UTRs of specific mRNAs and inhibit their translation or promote their degradation [23]. Viruses often exploit these microRNAs to augment their replication by targeting cellular antiviral proteins [24–26]. Here we report that mammalian microRNA, miR-24-3p (miR-24), is induced by Herpes simplex virus 1 by exploiting host cell kinases which facilitates viral replication. HSV1-induced miR-24 binds to the cognate site present in the 3’UTR of STING mRNA and inhibits its translation, dampening the antiviral response. Human cellular miR-24 is derived from the primary transcripts of two genetic loci: locus 1 on chromosome 9 and locus 2 on chromosome 19. In both loci, the transcripts include two other microRNAs, miR-23 and miR-27 clustered with miR-24. At locus 1, the C9orf3 gene encodes the mRNA of the AOPEP protein; its 3’UTR gives rise to miR-23b, miR-27b and miR-24-1 [27]. Locus 2 is transcribed to a non-coding RNA that contains precursors of miR-23a, miR-27a and miR-24-2 [28]. The transcriptional promoter of the C9orf3 gene contains a single binding site (–141 bp upstream to AOPEP gene), for the transcription factor AP-1, which is activated by MAP kinases. Talotta F. *et al* have experimentally verified this binding site (TAAGTCA) as potent AP-1 binding site [29]. The promoter region of locus 2 has been characterized between -603 to +36 bp [28] and no putative AP1 site was predicted upstream to locus 2 upto -800 bp (software PROMO was used for binding site prediction). HSV1-induced activation of MAP kinases triggers the synthesis of miR-24 to evade the antiviral action of STING. Consequently, virus replication in cell cultures and pathogenesis in mice could be attenuated by antimiR-24, which counteracts the action of miR-24.

## Results

### miR-24 targets the 3’UTR of STING mRNA

**Fig 1:**
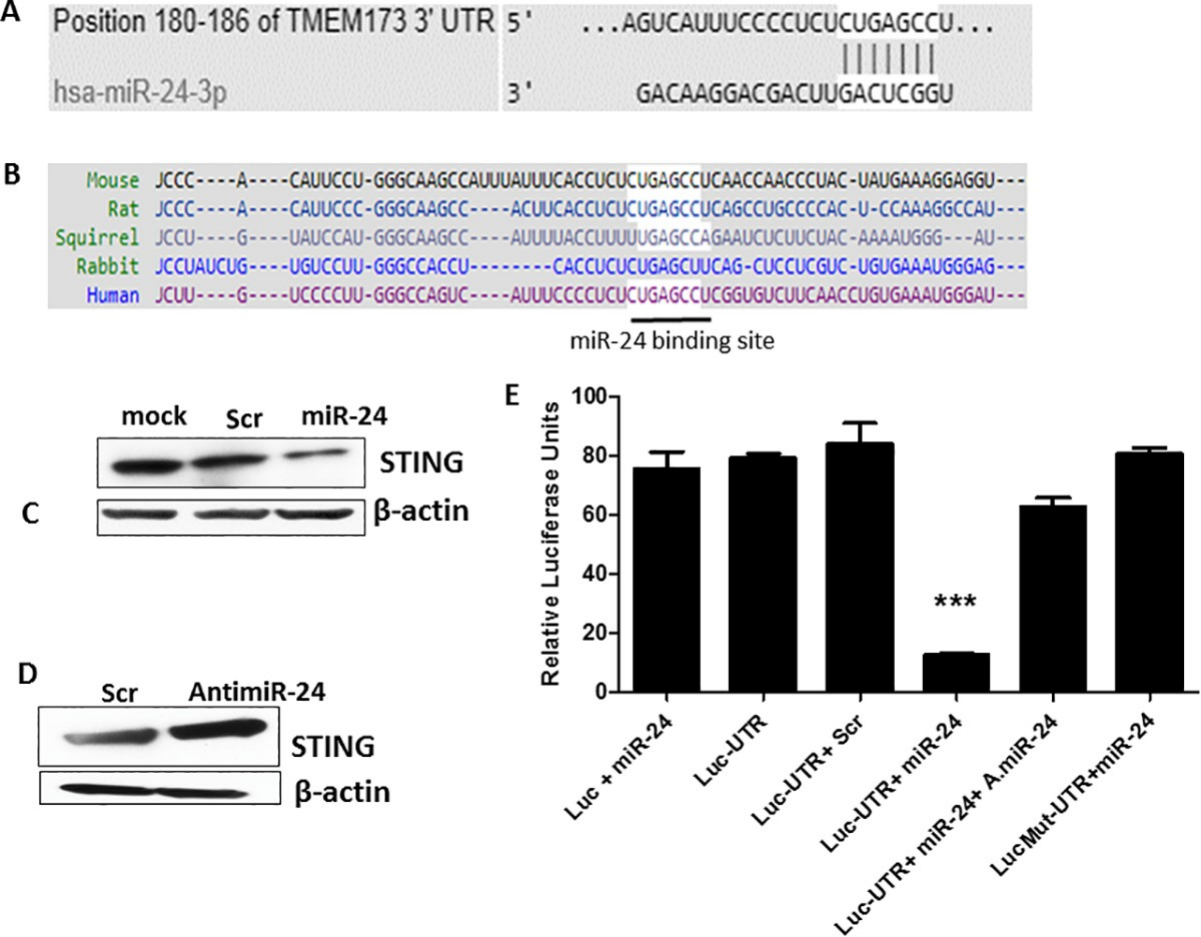
miR-24 targets 3’UTR of STING mRNA. **(A)** Bioinformatic softwares (Human Target scan, miRwalk) were used to identify microRNA-24 which targets 3’UTR of human STING mRNA at position 180 to 186. **(B)** Comparative analysis showing conserved miR-24 binding sites in STING 3’UTR across different species. **(C)** Human HT1080 cells were transfected with 1- Lipofectamine only, 2- Scramble control (300 pmol), 3- miR24 (300 pmol) and STING protein level was determined 36 hours post transfection by Western blotting. **(D)** HT1080 cells were transfected with 1- Scramble control for antimiR or 2- antimiR-24 and STING protein level was measured by western blotting 36 hours post transfection. **(E)** HeLa cells were transfected with 1- Luciferase expression vector+ miR-24, 2- Luciferase STING 3’ UTR vector, 3- STING 3’ UTR luciferase vector+ Scramble control, 4- Luciferase STING 3’ UTR vector + miR-24, 5- Luciferase STING 3’ UTR vector + miR-24+ antimiR-24, 6- Luciferase STING mutated 3’UTR vector + miR-24. The secretory luciferase activity in culture supernatant was measured and normalized by measuring secretory alkaline phosphatase activity. (**E**: Mean ± SEM, N = 3). *P<0.05, **P<0.01, ***P<0.001.

As viruses are known to exploit cellular microRNAs to target antiviral proteins, we looked for microRNAs that could target STING expression.by using various software (Human target scan, miRwalk), We observed that miR-24 has one complementary binding site on the 3’UTR of human STING mRNA (position 180–186 on 3’UTR) (Fig 1A). This binding site for miR-24 in STING mRNA is highly conserved in human, mouse and other species (Fig 1B); mouse STING mRNA 3’UTR has two potential binding sites at positions 49 and 139, which are identical in sequence to the site in the human STING mRNA. Huang *et al* reported miR-24 binding to rat STING mRNA [30]. Recently Shen *et al* and Khan *et al* have also reported this targeting in a liver ischemia model and Brucella infection respectively [31,32]. As anticipated, transfection of synthetic miR-24 caused a reduction of STING protein expression (Fig 1C); conversely, transfection of antimiR-24 enhanced STING expression (Fig 1D). Targeting of the 3’UTR of STING mRNA by miR-24 was validated by co-transfecting a luciferase-STING mRNA 3’UTR reporter vector (human STING mRNA vector) and miR-24, followed by luciferase assays; miR-24 inhibited luciferase expression by the reporter (bar 4, Fig 1E) and co-expression of antimiR-24 rescued it (bar 5, Fig 1E). Moreover, when the binding site of miR-24 in Luc-STING UTR was mutated, miR-24 could not inhibit luciferase expression anymore (bar 6, Fig 1E). The sequence of mouse and human miR-24 is identical, so we confirmed similar targeting of STING by miR-24 in mouse RAW cells too (S1A Fig). To study the effect of exogenous miR-24 expression on STING mediated gene induction, HT1080 cells were stimulated cells by transfecting cGAMP (activator of STING signaling); miR-24 suppressed IFN β mRNA induction whereas antimiR-24 augmented it (S1B Fig). Infecting cells with HSV1 triggers STING signaling which phosphorylates IRF3 and induces antiviral genes.

### HSV1 induced STING signaling is dampened by miR-24 by targeting STING

**Fig 2:**
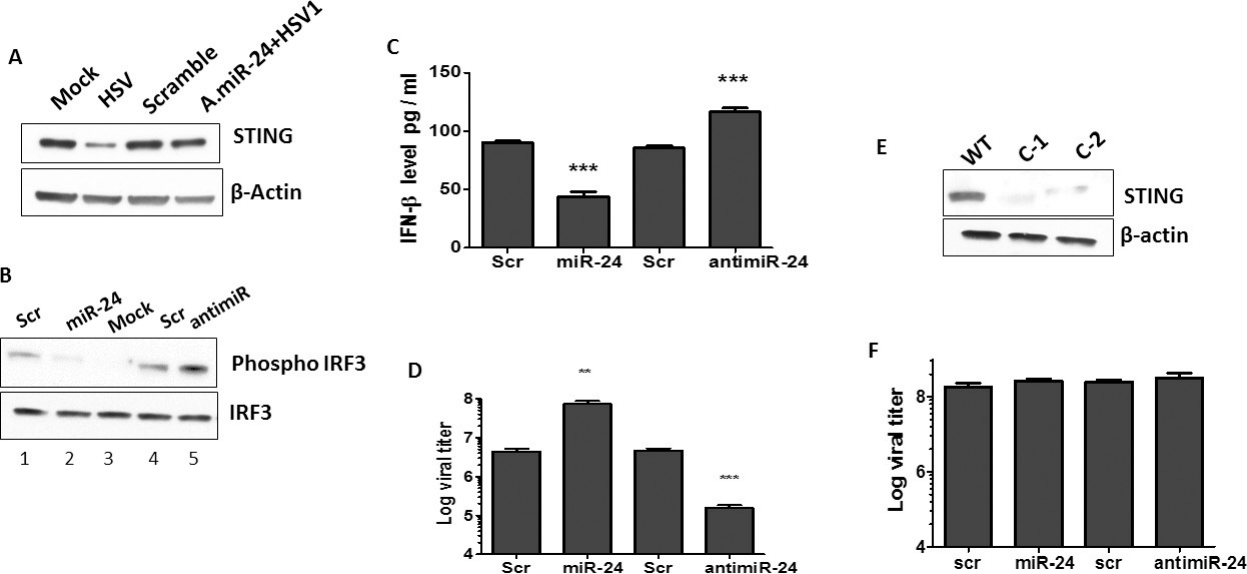
miR-24 promotes HSV1 replication by suppressing STING expression. **(A)** HT1080 cells were transfected with Scramble control (lane 3), or antimiR-24 (lane 4). 24 hours later, HSV1 infection was given to antimiR-24 transfected cells (lane4) and STING protein levels were determined after 16 hours post infection. Mock (lane 1) and HSV1 infected HT1080 cells (lane 2) (16hrs post infection) were also analyzed. **(B)**. HT1080 cells were transfected with 1-Scramble control, 2-miR-24, 3-mock control, 4- Scramble for antimiR 5- antimiR-24 and HSV1 infection was given 24 hours post transfection. Phospho-IRF-3 and IRF-3 levels were determined 3 hours post HSV1 infection. **(C)** HT1080 cells were transfected with 1-Scramble control, 2-miR-24, 3-Scramble control for antimiR or 4-antimiR24. HSV1 infection (MOI-5) was given 24 hours post transfection and Interferon-β protein in culture supernatants was quantified by sandwich ELISA 10 hours post HSV1 infection. **(D)** HT1080 cells were transfected with 1-Scramble control, 2-miR-24, 3-Scramble control for antimiR or 4-antimiR-24. HSV1 infection was given 24 hours post transfection and viral titer was quantified 12 hours post HSV1 infection. The cells along with culture media was frozen and thawed thrice to determine titer in cells and media. **(E)** STING expression was analyzed in WT and STING KD HT1080 cells (2 clonal isolates C1 and C2 were analyzed) to confirm knockdown of STING. **(F)** HT1080 STING knock down cells were transfected with 1-Scramble control, 2-miR-24, 3-Scramble control for antimiR or 4-antimiR-24. HSV1 infection was given 24 hours post transfection and viral titer in cells and culture supernatants was quantified 12 hours post HSV1 infection. (**C, D, F**: Mean ± SEM, N = 3). *P<0.05, **P<0.01, ***P<0.001.

HSV1 virus activates antiviral STING signaling leading to IRF3 activation and production of interferons. Because STING is the pivotal molecule controlling cellular innate immune response against DNA viruses, STING expression pattern in HSV1 infected cells was examined. HSV1 infection caused reduced expression of STING in HT1080 cells (Fig 2A). Prior transfection of miR-24 inhibited IRF3 activation in HSV1 infected cells, whereas antimiR-24 enhanced it (Fig 2B); consequently, IFN-β mRNA and IFIT-1 mRNA induction by virus infection was suppressed by miR-24 and augmented by antimiR-24 (S1C and S1D Fig). As expected, secretion of IFN-β from the infected cells to the culture medium was affected similarly; compared to control cells, less IFN was secreted by miR-24-transfected cells and more IFN-β was secreted by antimiR-24-transfected cells (Fig 2C). Hence, miR-24 dampened HSV1 induced STING signaling and the above effects were reflected by the efficiency of HSV1 replication. More virus was produced by the miR-24 transfected human cells whereas antimiR-24 transfected cells exhibited the opposite effect (Fig 2D). To ensure that the observed effects of miR-24 and antimiR-24 on virus replication was mediated by STING, we knocked down STING expression in HT1080 cells using CRISPR-Cas9. In two clonal isolates, STING expression was almost completely abolished (Fig 2E). Both clones produced similar results; miR-24 and antimiR-24 had no effect on HSV1 replication in cells devoid of STING expression (Fig 2F) and virus replicated more efficiently in these cells compared to the parental cells (compare bar 1, Fig 2D and bar 1, Fig 2E). These results indicated that the effects of miR-24 and antimiR-24 on virus replication are mediated entirely by their effects on STING. Similar phenomenon was observed in mouse cells transfected with miR-24 and antimiR-24 (S1E Fig). The results presented above demonstrated that endogenous STING expression can be manipulated by exogenous miR-24; which, in turn, can affect IFN induction and virus replication.

### MAPKs activated by HSV1 infection induce miR-24 and promote virus replication

**Fig 3:**
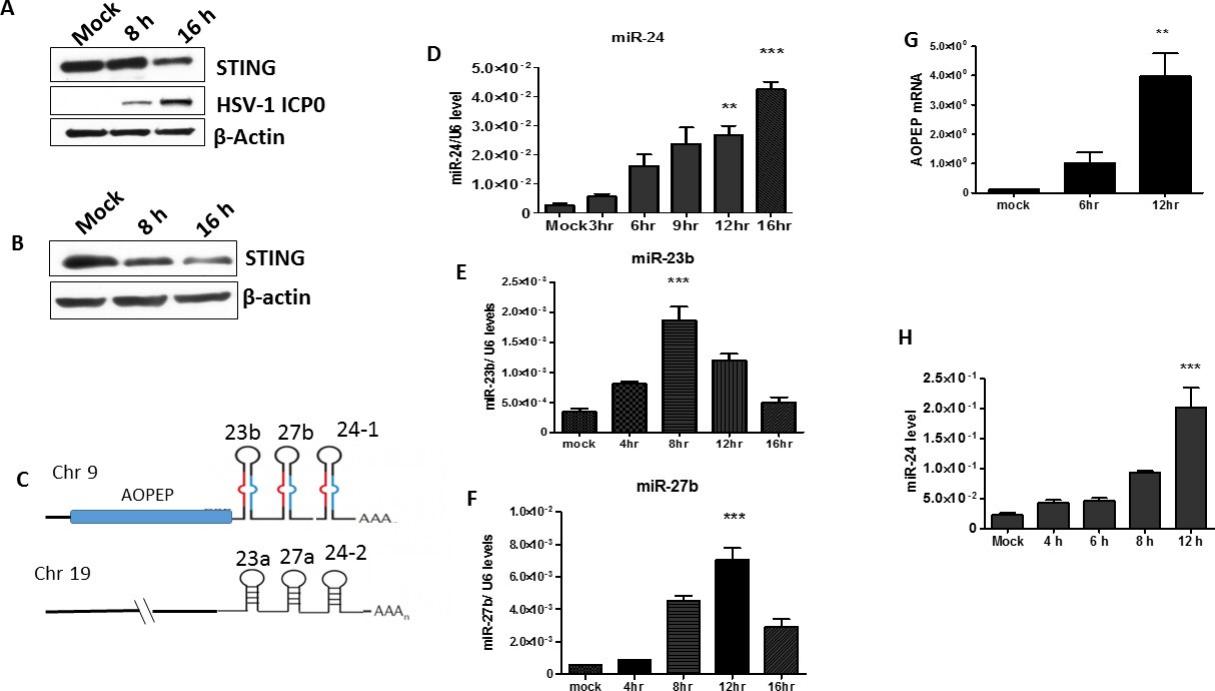
HSV1 infection induces expression of the AOPEP-miR-23b/ miR-27b/ miR-24-1 locus. **(A) (B)** STING expression was analyzed by western blotting in human (A) HT1080 cells and (B) mouse NB41A3 cells upon HSV1 infection (MOI-5). **(C)** Figure depicting expression of miR-24 from 2 genetic loci. mir-24 is expressed in clusters from human chromosome 9 (miR-23b/ miR-27b/ miR-24-1) and chromosome 19 (miR-23a/ miR-27a/miR-24-2). Chromosome 9 cluster is expressed along with AOPEP mRNA. Human HT1080 cells were infected by HSV1 and cells were harvested at various time points. **(D)** miR-24, **(E)** miR-23b and **(F)** miR-27b expression levels were measured. **(G)** The expression of AOPEP mRNA was quantified. **(H)** Mouse NB41A3 cells were infected by HSV1 and cells were harvested at various time points to determine miR-24 levels. (**D, E, F, G, H**: Mean ± SEM, N = 3). *P<0.05, **P<0.01, ***P<0.001.

Once we established that HSV1 replication can be experimentally regulated by miR-24 administration, we wondered whether the virus physiologically exploits this system to enhance its replication. Indeed, STING expression was repressed upon virus infection of human HT1080 cells and mouse NB41A3 cells (Fig 3A and 3B). Human miR-24-1 along with miR-23b and miR-27b, is embedded in the 3’UTR of AOPEP mRNA (Fig 3C); all were induced in HT1080 cells by virus infection (Fig 3D–3G). Mouse miR-24 was induced by HSV1 infection in NB41A3 cells as well (Fig 3H). Human miR-24-2, with the same sequence as miR-24-1, miR-23a and miR-27a originate from a non-coding transcript of a gene on chromosome 19 (Fig 3C). HSV1 did not induce the synthesis of miR-23a and miR-27a (S2A and S2B Fig). STING was not required for the induction of miR-24 by HSV1 (S2C Fig) and HSV1 infection in cells caused a reduction of STING expression (Fig 2A), an effect that was prevented by transfection of antimiR-24 prior to infection, confirming that miR-24, induced by the virus, was responsible for the observed reduction of cellular STING level and silencing of miR-24 prevented the reduction of STING. Neither HSV1 infection nor miR-24 transfection affected the expression levels of STING mRNA (S2D Fig). Because miR-27b has a putative binding site on the 3’UTR of STING mRNA [33], we tested its effect on STING translation by transfecting it into HT1080 cells; unlike miR-24, miR-27 did not inhibit STING synthesis (S1F Fig). Similarly, although miR-23 has been reported to inhibit cGAS synthesis in other cell types [34], virus infection of HT1080 cells did not cause a reduction of cGAS (S1G Fig). These results indicated that in our experimental system, only miR-24 affected the cGAS-STING signaling pathway.

**Fig 4:**
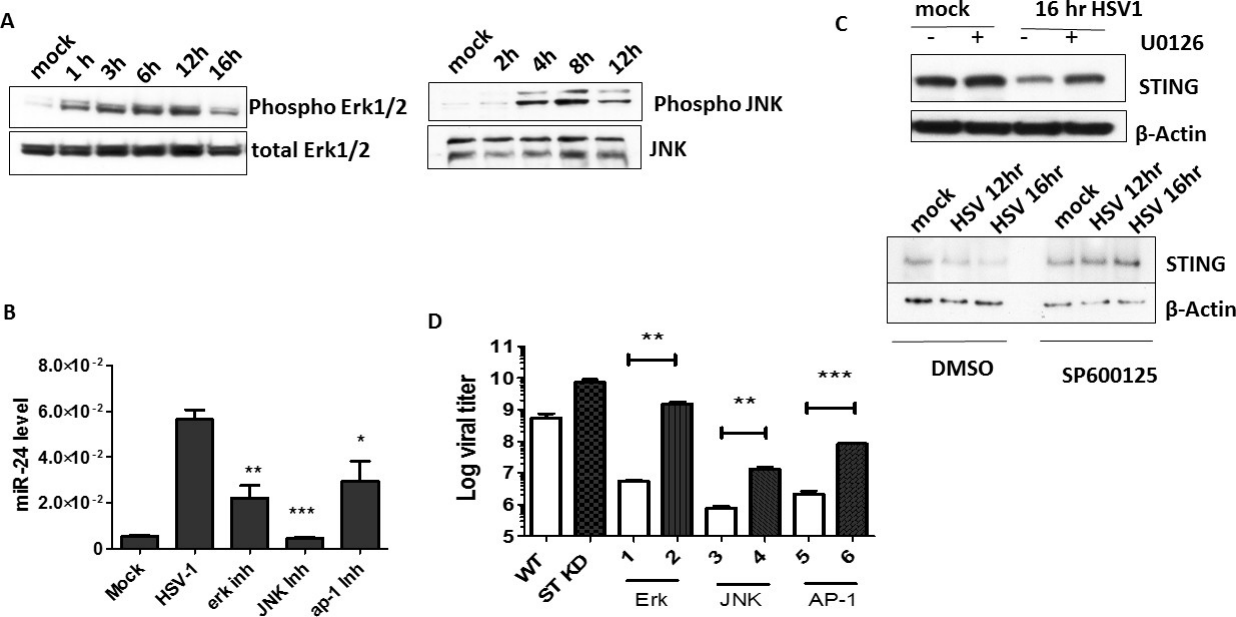
HSV1 infection activates MAPKs to induce miR-24 and promote viral replication. **(A)** HT1080 cells were infected with HSV1 and phosphorylation of Erk1/2 and JNK was determined by western blotting. **(B)** HT1080 cells were treated with DMSO, Erk inhibitor (U0126), JNK inhibitor (SP600125) or AP-1 inhibitor (t-5224) 2 hours prior HSV1 infection. The infected cells were incubated in presence of inhibitors (10μM) for 16 hours post infection and miR24 level was quantified. **(C)** HT1080 cells were pretreated with Erk inhibitor U0126 or JNK inhibitor SP600125 and HSV1 infection was given after 2 hrs. Cells were again treated with inhibitors 1hr post infection and were harvested to analyze STING expression after indicated time. **(D)** The viral titer in cells and culture supernatants of HT1080 and STING KD HT1080 cells infected by HSV1 in presence of various inhibitors (similar to (B)) was determined (Black bars indicate STING KD cells). The infected cells were incubated in presence of inhibitors (10μM) for 16 hours post infection and supernatant and cells were collected for viral titer estimation. (**B, D,—**Mean ± SEM, N = 3). *P<0.05, **P<0.01, ***P<0.001.

**Fig 5:**
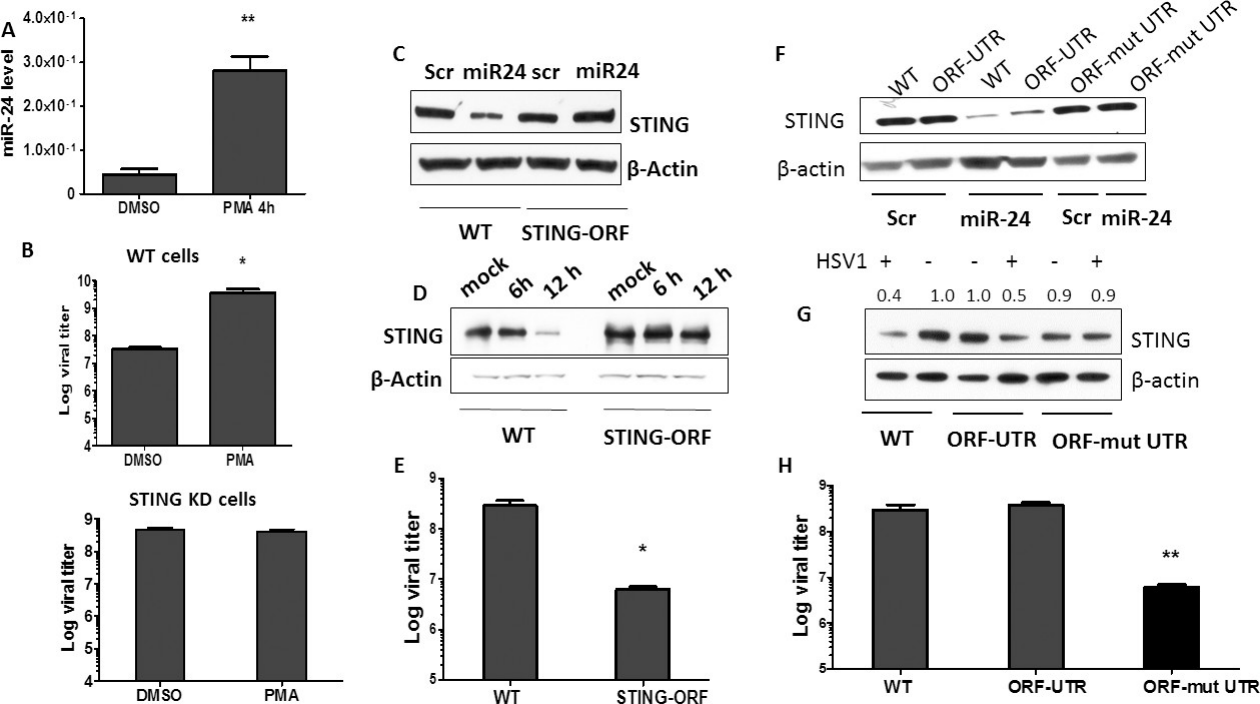
Pre induction of miR-24 by MAPK activator promotes viral replication and effect of miR-24 is regulated through 3’UTR of STING mRNA. **(A)** HT1080 cells were treated by PMA (10μM) and miR-24 expression was determined. **(B)** HT1080 cells or HT1080 STING KD cells were treated with PMA and then HSV1 infection was given 2 hours post treatment. HSV1 virus titer was quantified in cells and culture supernatants 8 hours post infection. Control group was treated with DMSO. **(C)** HT1080 cells and HT1080 STING KD cells reconstituted with STING-ORF were transfected by Scramble or miR-24 and cells were harvested after 24 hours to determine STING protein level by western blotting. **(D)** HT1080 cells and HT1080 STING KD cells reconstituted with STING-ORF were infected with HSV1 and cells were harvested after 6 and 12 hours to determine STING protein level by western blotting. **(E)** HT1080 cells and HT1080 STING KD cells reconstituted with STING-ORF were infected with HSV1, cells and culture media were harvested after 12 hours to determine viral titer. **(F)** HT1080 cells (WT), HT1080 STING KD cells reconstituted with ORF-UTR (containing STING ORF+3’UTR) and HT1080 STING KD cells reconstituted with ORF- mut UTR (containing STING ORF + mutated 3’ UTR where the binding site for miR-24 has been mutated in 3’UTR) were transfected with Scramble RNA or miR-24 and cells were harvested after 24 hours to determine STING protein level by western blotting. **(G)** HT1080 cells, HT1080 STING KD cells reconstituted with ORF-UTR (containing STING ORF+3’UTR) and HT1080 STING KD cells reconstituted with ORF- mut UTR (containing STING ORF + mutated 3’ UTR where the binding site for miR-24 has been mutated in 3’UTR) were infected with HSV1 and cells were harvested after 12 hours to determine STING protein level by western blotting. (Numbers above the blot represent densitometric quantification of STING normalized against actin) **(H)** HT1080 cells, HT1080 STING KD cells reconstituted with ORF-UTR and HT1080 STING KD cells reconstituted with ORF-mut UTR were infected with HSV1 and cells and culture media were harvested after 16 hours to determine viral titer. (**A, B, E, H—**Mean ± SEM, N = 3). *P<0.05, **P<0.01, ***P<0.001.

Next, we investigated the mechanism of miR-24 induction by HSV1 infection. Transcription of the gene encoding miR-24-1 (AOPEP gene) is driven by the Erk1/2 activation [35], which is a Mitogen Activated Protein Kinase (MAPK); therefore we inquired whether HSV1 induces miR-24 by activating MAPKs. Both ERK1/2 and JNK MAPKs were activated rapidly after HSV1 infection, as evidenced by their phosphorylation in HT1080 cells (Fig 4A) as well as in NB41A3 cells (S2F Fig) and virus infection activated c-Jun and c-Fos required for AP-1 activation (S2E Fig). HSV1 induced MAPK activation did not require STING (S2G Fig). More importantly, pharmacological inhibitors of ERKs, JNK and AP1, all strongly inhibited miR-24 induction by the virus (Fig 4B) and both Erk and JNK inhibitors prevented the decline of the expression of STING post virus infection (Fig 4C). To assess the effect of MAPK activation on overall virus replication, we measured virus replication in cells treated with the same inhibitors. To attribute these effects specifically to miR-24 mediated regulation of STING expression, we compared cells expressing or not expressing STING (Fig 4D). As expected, HSV1 replicated better in STING knock-down (ST KD) cells than in WT cells. The ERK inhibitor strongly inhibited virus replication in WT cells but had only a minor effect on ST KD cells, indicating that miR-24 mediated STING regulation plays a major role in HSV1 replication. JNK and AP1 inhibitors affected virus replication in both cell types, presumably because they also interfered with STING-unrelated pathways that help the process of viral replication. But, nonetheless, in all cases, virus replication was strongly impaired by these inhibitors and the impairment was, at least partly, mediated by STING. To further demonstrate the important role of MAPKs in miR-24 mediated regulation HSV1, cells were treated with PMA (Phorbol 12-myristate 13-acetate), a known activator of MAPKs; both Erk and JNK were activated (S3A Fig), miR-24 was induced (Fig 5A) and cellular STING level was lowered (S3B Fig). HT1080 cells treated with PMA induced miR-24 (Fig 5A), miR-23b and miR-27b but not miR-23a and miR-27a (S3C–S3F Fig). As expected, PMA pre-treatment enhanced virus replication in WT, but not STING KD cells (Fig 5B). These results confirmed that the induction of miR-24, due to activation of MAPKs and AP-1 transcription factors, regulated STING levels and viral replication.

### HSV1 replication is less efficient in cells expressing miR-24-unresponsive STING mRNAs

To confirm the mechanism of miR-24-mediated inhibition of STING synthesis, we designed a STING mRNA expression vector, which does not include its 3’UTR, so that miR-24 cannot bind and exert its effect on STING synthesis. STING KD cells were transfected with this expression vector and cell clones were isolated; a clone expressing STING at a level similar to that in WT cells (Fig 5C, lanes 1 and 3) was selected for further analysis. Transfection of miR-24 reduced the level of STING expression in WT cells, but not STING 3’UTR minus reconstituted cells (Fig 5C, lanes 2 and 4). In the latter cells, STING expression was hardly changed with progression of HSV1 infection. In contrast, STING expression was much reduced after infection of WT cells (Fig 5D) consequently, virus replication was more robust in WT cells (Fig 5E). These results demonstrated that HSV1 regulates cellular STING expression primarily through the 3’UTR of its mRNA and this regulation has major effects on the efficiency of virus replication. To further define the above conclusions, additional STING expression vectors were designed; these expressed mRNAs containing the STING coding sequence (ORF) plus the wild type 3’UTR (ORF-UTR) or the UTR in which the miR-24 binding site had been mutated (ORF- mut UTR); STING KD cells were reconstituted with these expression vectors. Transfected miR-24 inhibited STING synthesis in both WT HT1080 cells and cells expressing ORF-UTR, but not in those expressing ORF-mut UTR (Fig 5F). Similarly, HSV1 infection inhibited STING expression in WT and ORF-UTR cells, but not ORF-mut UTR cells (Fig 5G). Consequently, virus replication was inhibited only in ORF-mut UTR cells (Fig 5H). These results firmly established an exclusive role of miR-24 in the 3’UTR mediated effect of HSV1 on STING mRNA translation.

### Administration of antimiR-24 inhibits HSV1 replication *in vivo* and reduces morbidity and mortality of infected mice

**Fig 6:**
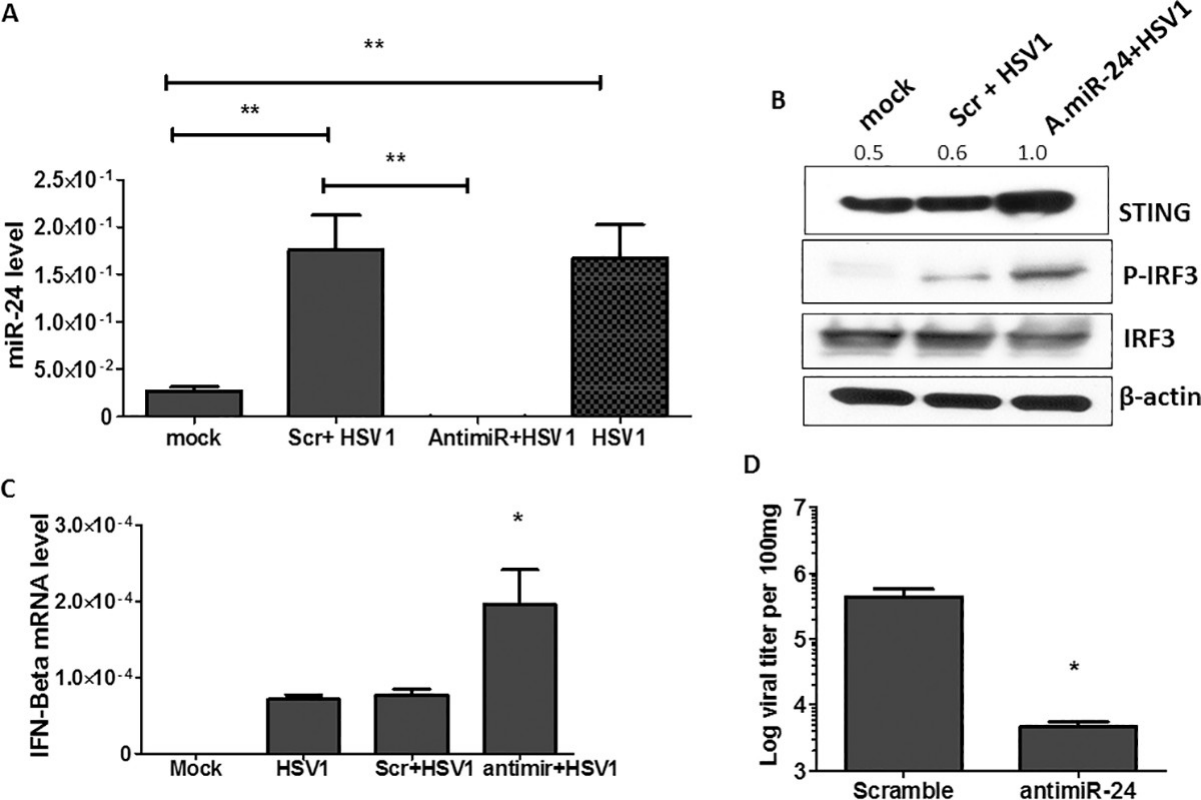
Administration of antimiR-24 inhibits HSV1 replication *in vivo* and enhances antiviral response in mouse brain. 1nmol of antimir-24 or scramble control for antimiR mixed with HSV1 (10^4^ PFU) was injected into 6 weeks old male mice intracranially on the left side of cranium. PBS injected mice and only virus injected mice were used as control. The brains were isolated 4 days post infection and whole brain was homogenized. Fractions were separated for RNA quantification, western blotting and viral titer assay from homogenized brain. (A) miR-24 expression was analyzed in brain samples. (B) STING, IRF-3 phosphorylation and total IRF-3 levels were analyzed from scramble + HSV and antimiR-24 + HSV (10^4^ PFU, day 4 post infection) administered mouse brains. (The numbers above the blot represent normalized densitometric analysis of STING normalized against actin) (C) RNA was isolated from brain tissue of infected mice (10^4^ PFU, day 4) and expression of IFN-β mRNA was quantified. (D) Viral titer from brains of scramble or antimir-24 mixed with HSV1 (10^4^ PFU, day 4) administered mice was determined. (A, C, D Mean ± SEM, N = 4). *P<0.05, **P<0.01, ***P<0.001.

**Fig 7:**
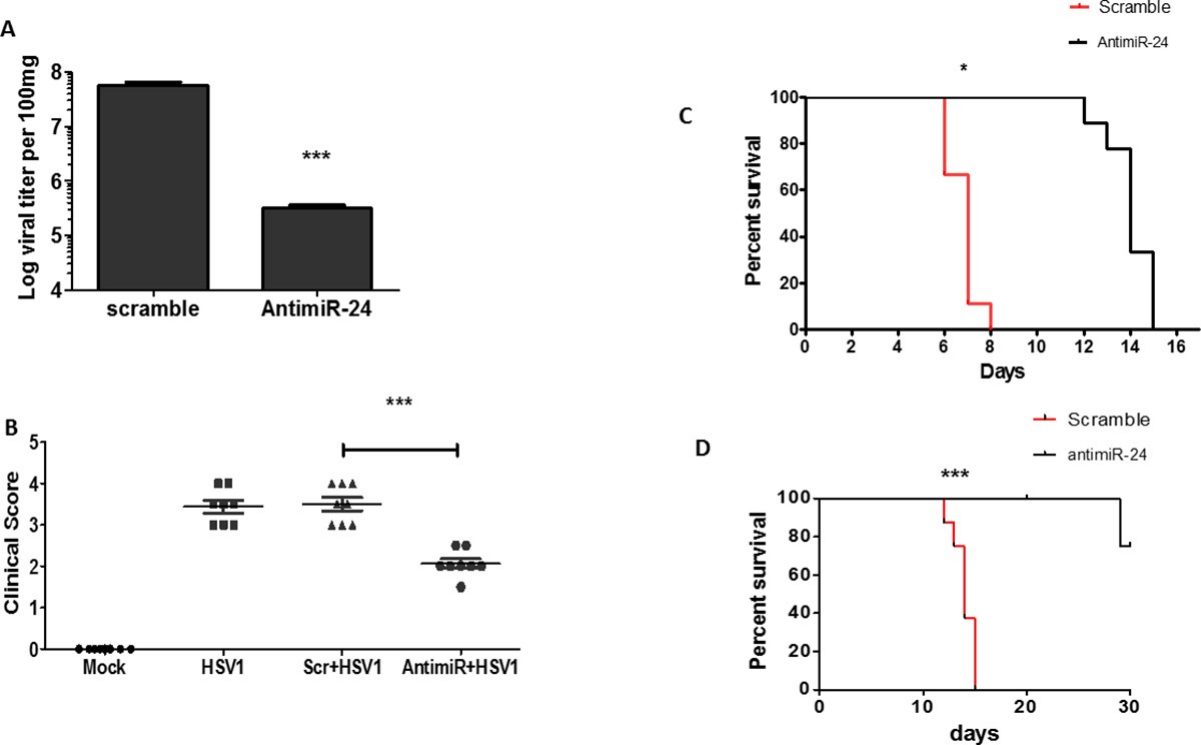
Administration of antimiR-24 alleviates mortality of HSV1 infected mice. **(A)** 6 weeks old male mice were given HSV1 infection intraperitoneally (10^7^ PFU) and after 3 days, antimir-24 or scramble control for antimiR (1nmol) was injected into cranium. Brain was harvested 5 days after antimiR-24 injection and viral titer was determined (Mean ± SEM, N = 4). **(B)** The clinical neurological symptoms were observed in intracranially infected mice (given virus mixed with scramble control for antimiR or antimiR-24) on day 5 post infection and clinical score was given to various groups on basis of severity of disease. (Mean ± SEM, N = 8) **(C)** The survival curve analysis was done for mice intracranially injected with antimiR-24 or scramble control mixed with HSV1 (10^4^ PFU). **(D)** Low dose of HSV1 (500PFU) mixed with scramble control for antimiR or antimiR-24 was intracranially injected into mice and survival curve analysis was done. (**C, D** Mean ± SEM, N = 9). *P<0.05, **P<0.01, ***P<0.001.

After establishing that miR-24 plays a major role in HSV1 replication in cell cultures, we were interested to learn whether the same is true for virus infection in mice and whether experimental manipulation of miR-24 action can affect viral pathogenesis. To pursue these goals, we administered antimiR-24 or scramble RNA to infected mice and monitor the effect. These microRNA inhibitor oligonucleotides use LNA technology with phosphorothioate (PS)-modified backbones for enhanced stability and are quickly absorbed by the cells. Because HSV1 is a neurotropic virus, we chose to inject antimiR-24 intra-cranially. In one experimental model, HSV1 was injected, along with antimiR-24, intra-cranially. The virus induced miR-24 strongly in the brain and the degree of induction was very similar in the brains of infected mice which also received the scramble RNA. In contrast, miR-24 level was very low in the brain of infected mice that received antimiR-24 (Fig 6A). HSV1 induced miR-24 expression caused reduced STING protein expression in the brains of the infected mice (S4A Fig), STING expression was higher in the brains of infected mice administered with antimiR-24 than in the brains of those that received scramble RNA or mock infected (Fig 6B); more IRF3 was phosphorylated (Fig 6B) and more IFN-β mRNA (Fig 6C), Ifit1 mRNA (S4B Fig) and Ifit2 mRNA (S4C Fig) were induced in the brains of mice that received antimiR-24. More importantly, antimiR-24 administration caused strong inhibition of HSV1 replication as well (Fig 6D). In another model of pathogenesis, a much higher dose of virus was injected intraperitoneally and after three days antimiR-24 was injected intra-cranially; antimiR-24 strongly inhibited virus replication in this model as well (Fig 7A). We monitored morbidity of the intra-cranially infected mice by recording their clinical scores in several neurological functions and observed that antimiR-24 efficiently alleviated their symptoms (Fig 7B). Consequently, death of the infected mice was much delayed by antimiR-24 (Fig 7C). Similar beneficial effects of antimiR-24 on mouse survival was also observed when a 20 fold lower dose of virus was injected intra-cranially (Fig 7D). The above results demonstrated that HSV1 replication and resultant pathogenesis in mice are highly facilitated by miR-24; moreover administration of a single dose of antimiR-24 has a robust protective effect.

## Discussion

**Fig 8:**
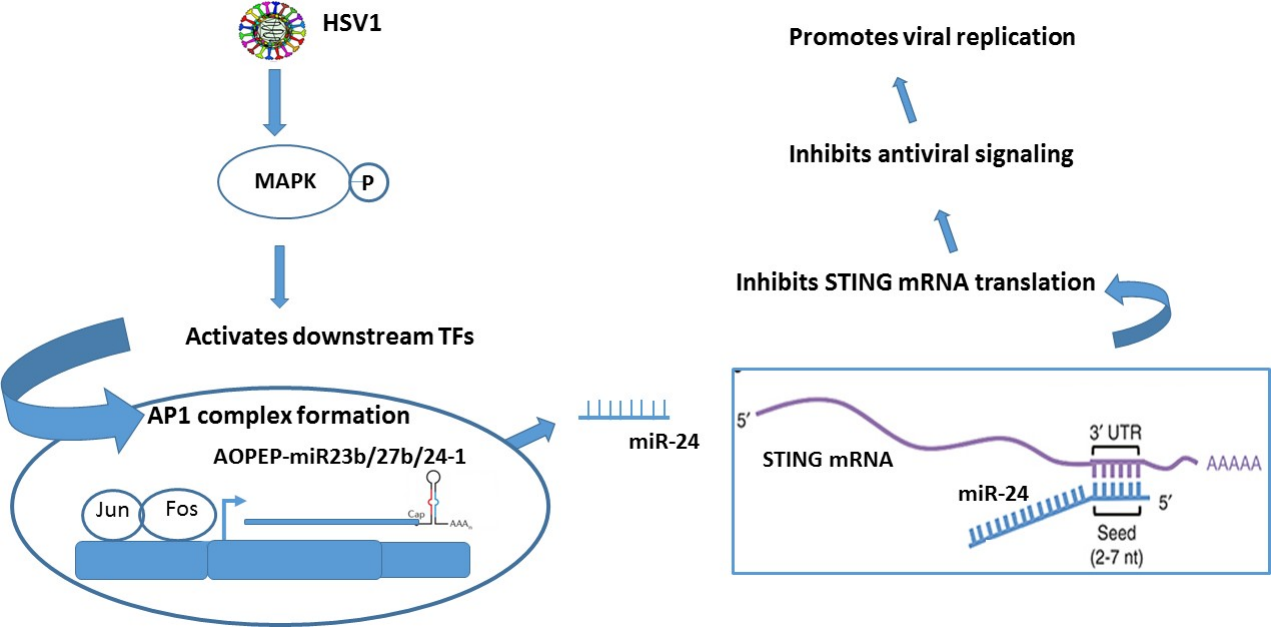
Model summarizing the regulatory role of miR-24 in HSV1 induced STING signaling. The model depicts the regulation of STING protein levels by HSV1 induced miR-24 expression which inhibits STING signaling and promotes viral replication. HSV1 infection activates MAPKs which enhance the expression of AOPEP mRNA and miR-23b, miR-27b, miR-24 leading to miR-24 accumulation and targeting of STING signaling. This acts as an immune evasion strategy adopted by the virus to augment its own replication.

Our findings, reported here, are presented schematically in Fig 8 depicting a positive feed-back loop induced by HSV1 to promote its replication. HSV1 is known to activate STING signaling, which then inhibits virus replication through both IFN-dependent and independent pathways [36–38]. We observed that HSV1 can evade STING’s antiviral effects by inhibiting its synthesis through the action of a microRNA, miR-24, which inhibits STING synthesis by binding to its cognate site in the 3’ UTR of STING mRNA. Inhibition of STING synthesis by virally induced miR-24 leads to less IFN induction and consequent enhancement of virus replication. Targeting of STING by miR-24 has been reported previously [30,31]. This miRNA can arise from two genetic loci (Fig 3C) and from both loci, its expression is accompanied by the expression of two other miRNAs, miR-23 and miR-27. We presented evidence that in our experimental system, expression of the three miRNAs was induced only from the C9orf3 gene in locus 1 on human chromosome 9. This genetic locus encodes AOPEP mRNA and the three miRNAs, miR-23b, miR-27b and miR-24, are embedded in its 3’UTR [27]. HSV1 infection induced synthesis of this transcript and production of mature AOPEP mRNA and the three miRNAs (miR-23b, miR-27b, miR-24). It has been reported that expression of miR-24 is regulated by Erk1/2 kinases and Erk inhibitors reduce the expression of c9orf3 mRNA [35]. Transcriptional induction of the C9orf3 gene was mediated by the transcription factor AP1, which has a cognate binding site in its promoter region; no such AP1 binding site was recognizable in locus 2 and viral infection did not induce expression of a transcript from this locus. Infection by HSV1 causes activation of the MAP kinases, ERK and JNK, which activate AP1 [39,40]; using pharmacological inhibitors we demonstrated that this pathway is used by the virus to induce miR-24.

HSV1 exploits many cellular proteins, including protein kinases, to support viral replication and pathogenesis in the host [41,42]. The mechanisms adopted by the virus contribute to its immune evasion strategy by successful hijacking of the host cell machinery [43]. It enhances the phosphorylation and activation of cellular MAPKs, which are essential for completing the viral replication cycle. HSV1 ICP27 proteins enhance phosphorylation of JNK and p38 MAP kinases required for viral replication [44]. Activation of Erk1/2 by HSV1 has also been reported to be crucial to viral replication [40]. Moreover, activation of JNK kinases is indispensable for reactivation of the virus from latency [45]. Here, we uncovered a novel example of pro-viral actions of MAPKs, activated in the HSV1 infected cell, through the induction of a cellular microRNA which aids viral replication.

MicroRNAs regulate cellular protein synthesis by binding to complementary sequences present in specific mRNAs, often in their 3’ UTRs. MicroRNA binding inhibits translation of the target mRNAs or promotes their degradation, the first effect being the predominant one in mammalian cells [46]. Several prediction software (Human target scan, miRwalk) indicated that miR-24 can bind specifically to the 3’UTR of STING mRNA. Its complementary binding sequence was perfectly conserved in both human and mouse STING mRNAs. We experimentally confirmed that HSV1-induced miR-24 inhibited STING synthesis using the cognate binding site in the 3’UTR of STING mRNA; if the 3’UTR was removed or the miR-24 binding site was mutated, STING mRNA translation was unaffected by miR-24 (Fig 5). Our results also indicated that STING 3’UTR is not targeted by other virus induced miRNAs, because STING expression levels were similar in infected and uninfected cells that expressed the STING mRNA with a mutation of the miR-24 binding site in the 3’UTR (the two rightmost lanes in Fig 5G). Indeed, miR-27b, which was induced along with miR-24 by HSV1 infection and has a putative binding site in the 3’UTR of STING mRNA [33], did not inhibit STING synthesis in HT1080 cells (S1F Fig). Yu *et al* [34] have reported that HSV1 suppressed miR-23 expression enhancing cGAS expression in mouse L929 cells. However, we did not observe any change in cGAS level upon HSV1 infection of HT1080 cells (S1G Fig). It appears that some effects of these miRNAs are cell type specific. miRNA-mRNA binding in cells is influenced by other regulatory factors (RNA binding Proteins, miRNA sponges) causing such differences in miRNA-mRNA interaction in various cell lines [47–49]. The effect of miR-24 on STING signaling is independent of cGAS, as experiments with STING agonist cGAMP also depicted the regulatory role of miR-24 on STING signaling (S1B Fig). Hence we can confirm that targeting of STING by HSV1 is mediated through miR-24 in HT1080 cells.

miR-24 has been known for its role in tumor progression [50], cell proliferation [51] and apoptosis [52]. Here we report the pro-viral role of miR-24 in facilitating HSV1 replication.

Viruses often use cellular or virally coded miRNAs to facilitate their replication [25]. For example, cellular miR-122 is essential for HCV replication in hepatocytes and antimiR-122 (Miravirsen), targeting this miRNA, is under clinical trials as a potential drug for alleviating HCV pathogenesis [53]. Recently Duygu *et al* have predicted miRNAs targeting the SARS-CoV-2 viral genome, which could be potentially exploited to target viral replication [54]. Similarly, miRNA-200c has been reported to target ACE2 mRNA, which encodes the receptor for SARS-CoV-2 cellular entry [55]. Our discovery of HSV1 induced miR-24 targeting STING synthesis adds to this list.

HSV1 takes advantage of multiple strategies to evade the antiviral effects of the IFN system [56]. None of these strategies are capable of total elimination of IFN’s actions; however, their cumulative actions are quite effective in helping virus replication [57]. IFN induction is inhibited by viral ICP27, UL42, UL46 and US3 proteins [58–60]. IFN signaling is inhibited by UL13, UL36 and ICP27 [61,62]. The cGAS/STING pathway is blocked by a variety of viral proteins: UL41 causes degradation of cGAS mRNA [63], γ_1_34.5 prevents translocation of STING from the ER to the Golgi [64], VP22 binds to cGAS whereas UL37 deamidates it, both inhibiting its enzyme activity [22,65]. Our study demonstrates that activation of MAPKs by virus infection induces the synthesis of miR-24 which inhibits STING synthesis.

HSV1 may have different effects on STING expression in different cell lines. HSV1 infects mucosal epithelium and then resides in neuronal ganglions. For our studies, we chose human HT1080 cells as they are epithelial in origin and express very low basal level of miR-24. These cells have good expression of STING and can easily sustain HSV1 infection without showing any cell death up to 16hrs post infection. Mouse NB41A3 neuroblastoma cells are neuronal in origin and expressed very low basal level of miR-24. So, they were used in our study of HSV1 infection of neuronal cells, a prelude to our in vivo experiments. In both cell lines, we observed decreased STING expression upon HSV1 infection. However studies by Bodda *et al* reported that HSV1 infection of THP1 cells enhanced STING levels and an opposite effect was observed in infected mouse bone marrow derived macrophages, indicating cell type specific regulation of STING by HSV1 [66]. Because MAPKs are activated by many cytokines and a variety of cellular stresses, miR-24-mediated inhibition of STING synthesis is likely to be used widely to regulate STING functions in other microbial infections, aberrant DNA metabolism and cancer. Indeed, when our manuscript was in preparation, Khan *et al* reported a role of miR-24 in promoting replication of Brucella, through inhibition of STING synthesis [32].

Our experimental results indicated that miR-24-mediated inhibition of STING synthesis plays a major role in the efficacy of HSV1 replication. Pre-induction of miR-24 before virus infection impairs virus replication whereas blocking its induction by the virus, using MAPK inhibitors, enhances virus replication. Moreover, in cells expressing a STING mRNA devoid of the miR-24-binding site, HSV1 replicates poorly. We demonstrate that HSV1 replication is manipulated by administration of miR-24 or antimiR-24 in human HT1080 and mouse RAW cells. Encouraged by previous demonstrations of activities of other antimiRs *in vivo* [67,68], we tested for similar effects of antimiR-24. Intra-cranial injection of a single dose of this RNA was very effective in reducing morbidity and mortality of HSV1-infected mice. Unexpectedly, miR-24 expression in the brains of infected mice was strongly inhibited by the administration of antimiR-24 (Fig 6A), which probably caused the enhanced expression of STING (Fig 6B). Strikingly, antimiR-24 was effective even when administered three days after peripheral infection of the mice. These results indicate possible therapeutic use of stable anti-miR-24, albeit administered by other routes, in diseases that are alleviated by STING. miR-24 is known to affect brain development and fetal neuronal differentiation [69]; however these effects should have little bearing on our experimental system, because we used young adult mice and miR-24 expression was only transiently affected by virus infection and antimiR-24 administration.

## Materials and methods

### Ethics statement

All animal experiments were performed in compliance with protocols approved by the Cleveland Clinic Institutional Animal Care and Use Committee.

### Reagents and antibodies

We purchased from cell signaling technology (CST) antibodies for STING (13647S), actin (3700, CST), phospho Erk1/2 (9101S, CST), total Erk (4695S CST), cGAS (15102S) phosho JNK (9251S CST), total JNK (9252S CST), phospho c-Jun (9261S, CST), total c-Jun (9165S, CST), phospho c-Fos (5348S, CST), total c-Fos (4384S, CST), phospho IRF3 (4947S, CST), total IRF3 (4302S, CST) and HSV1 ICP0 antibody (sc-53070) from Santa Cruz biotechnology. Lipofectamine 2000 (Invitrogen) was used for cGAMP (cat no- tlrl-nacga23-5, InvivoGen) transfection. For knockdown of STING in HT1080 cells, LentiCRISPRv2 vector (addgene 98291), psPAX2 vector (addgene 12260) and p-VSVg (addgene 8454) vectors were used. STING-ORF plasmid used for STING reconstitution in HT1080 cells was cloned in our laboratory in pcDNA3.1 (+) vector. The vector was transfected into HT1080 cells and neomycin selection was used to generate stable cell lines. For mouse brain frozen tissue homogenization, PARIS kit (cat no- AM1921 Thermo Fisher Scientific) was used to isolate RNA and protein. Luciferase STING 3’UTR vector (HmiT100627-MT05) and control luciferase vector (CmiT000001-MT05) was purchased from genecopoeia.

MAPK activator PMA (P1585 Sigma), ERK inhibitor U0126 (tlrl-u0126, InvivoGen) and JNK inhibitor SP600125 (tlrl-sp60, InvivoGen) were used for MAPK inhibitor experiments. AP-1 inhibitor t-5224 was kind gift from Dr. Christine O’Conner’s laboratory.

### STING ORF-UTR vector cloning and mutagenesis

STING ORF-UTR plasmid was cloned in our laboratory in pLVX vector. STING-ORF vector was amplified by using high fidelity polymerase (KOD hot start DNA polymerase, #71086–3, Millipore sigma) using inverse PCR method and fused to amplified STING 3’UTR (ClonExpress II One Step Cloning Kit, #C112, Cellagen Technology). The vector was transfected into HT1080 cells and puromycin selection was used to generate stable cell lines. The STING ORF-UTR vector was then used as template to mutate miR-24 binding site (CTGAGCC to TACTCAA) by using specific primers (ORF-Mut UTR vector). The vector was amplified by using specific primers for mutation by using high fidelity polymerase and ligated by using T4 ligase (#171093, New England Biolabs). For mutating miR-24 binding sites in luciferase 3’UTR vector, same primers were used for amplification (inverse PCR) of luciferase 3’ UTR vector creating mutation in miR-24 binding site and linear amplified vector was ligated. The list of primers used has been provided in S1 Table.

### MicroRNA mimics, inhibitors and transfection

miRNA mimics are chemically modified double-stranded RNA molecules designed to mimic endogenous microRNAs (miRNAs) which work similar to endogenous microRNAs by binding host mRNAs. miR-24 mimic (cat no. 4464066, Assay i.d- MC10737), miR-23a mimic (4427975 Assay i.d- 000399), mir-23b mimic (4427975 Assay i.d- 000400), miR-27a mimic (4427975 Assay i.d- 000408), miR-27b mimic (4427975 Assay i.d- 000409), Negative Control for mimic (cat no. 4464058), miRNA inhibitor for mir-24-3p (cat no. 4464084, assay i.d MH10737), Anti-miR miRNA Inhibitor Negative Control (Cat no- AM17010) were purchased from Thermo Fisher Scientific. For miR-24 inhibition in mice brain, LNA power miRNA inhibitor (cat no.-339147 YCI0200961-FFA) and Negative control (339147 YCI0201821-FFB) oligonucleotides were customized from Qiagen. These LNA inhibitors use LNA technology with phosphorothioate (PS)-modified backbones for enhanced stability. For microRNA overexpression and inhibition, 300 pmol oligos were transfected into HT1080 cells using DharmaFECT 4 transfection reagent (GE Lifesciences) following manufacturer’s instructions. For microRNA quantification, Taqman microRNA assay for specific to particular microRNA and RNU6 control (4427975, assay i.d 001006) were used along with Taqman microRNA reverse transcriptase kit (cat no.- 4366596) and Taqman Universal RT-PCR master mix (cat no- 4304437). The sequence of all microRNA mimics and inhibitors is provided in S1 Table

For cGAMP experiments, HT1080 cells were transfected with 300pmol miR-24 or antimiR-24 (using DharmaFECT 4) and after 24h cells were transfected with 8 μg/ml of cGAMP using lipofectamine 2000 following manufacturer’s instructions. 1h later, medium was removed and fresh medium was added. Cells were harvested after 8 hours post cGAMP treatment.

### Cell culture

All cell lines were maintained in DMEM supplemented with 10% FBS, 100 units/ml of penicillin, and 100 mg/ml of streptomycin. HT1080 WT (CCL-121), Vero cells and NB41A3 cells (CCL-147) were obtained from ATCC (Manassas, VA, USA). HT1080 STING KD cells were generated in our laboratory by using CRISPR/Cas9 technology. Human STING sgRNA sequence: GGTGCCTGATAACCTGAGTA was used. LentiCRISPRv2 vector containing guide RNA, psPAX2 plasmid and p-VSVG plasmid (psuedotyping plasmid) was transfected into 293T cells to generate lentiviral particles. The cell supernatant containing lentiviral particles was collected after 48 hrs and was used to infect HT1080 cells. Infected cells were grown with hygromycin in media as selection marker. After drug selection, single cell clones were sequenced and then screened for maximum STING deficiency using Western blot.

### HSV1 infection and virus quantification

HSV1 (KOS) propagation was performed as previously described (Blaho *et al*., 2005). Human HT1080 cells and mouse neuroblastoma cells NB41A3 cells were infected by HSV1 (MOI-5) for 1hour and then medium was replaced by fresh media. MOI-5 was used for all virus infection experiments. For viral titer quantification, the cells along with culture supernatant were frozen and thawed thrice to lyse the cells and then the supernatant was collected for titer assay. TCID50 assay of was performed (Spearman-Kärber method was used) to determine viral titer.

For microRNA experiments with HSV1 infection, Cells were first transfected with mir-24 mimic or scramble (300pmol) and 24 hours later HSV1 infection was given to cells. Later the cells were harvested 8hrs post infection for analyzing IFN-β and IFIT mRNA expression. For ELISA, cells were incubated 10 hours post infection. For viral titer assays, culture supernatant was collected 16 hours post infection.

### MAPK inhibitor treatment

10μM Erk inhibitor U0126, JNK inhibitor SP600125 and AP-1 inhibitor T-5224 were treated to HT1080 cells for 2 hours, then cells were infected by HSV1 and after 1 hr, media was again added with inhibitors. Cells were harvested and culture supernatant was collected 16hrs post infection for viral titer determination.

For MAPK activation, 10μM MAPK activator PMA was pretreated 2 hrs prior infection and later HSV1 infection was given to cells and cells along with culture supernatant was collected 8 hours post infection for viral titer determination.

### miRNA quantification, mRNA quantification and western blotting

For miRNA and RNA isolation, miRNeasy mini kit (Qiagen) was used according to manufacturer’s protocol. Further cDNA synthesis was done using reverse transcriptase kit (Thermofisher scientific). For real-time PCR, 384 well-format real time PCRs in a Roche LightCycler 480 II using Applied Biosystem’s SYBR Green PCR core reagents. For miRNA quantification, Taqman reverse transcriptase kit was used along with primer probe specific for miR-24-3p, miR-23a/b, miR-27a/b or RNU-6 (normalization control). Taqman master mix was used for RT-PCR. The sequence of primers used for RT-PCR is provided in S1 Table.

For mice brain tissue, the frozen tissue was homogenized and a fraction from the whole lysate was then used to isolate RNA following manufacturer protocol.

For western blotting, cells were lysed in lysis buffer containing 20 mM Hepes (pH 7.4), 50 mM NaCl, 1.5 mM MgCl_2_, 2 mM dithiothreitol (DTT), 2 mM EGTA, 10 mM NaF, 12.5 mM β-glycerophosphate, 1 mM Na_3_VO_4_, 5 mM Na-pyrophosphate, 0.2% (v/v) Triton X-100, and protease inhibitors (Roche Applied Science). The samples were boiled with 4X laemmli buffer and run on SDS-PAGE gel. The protein was transferred to PVDF membrane and blocked in 5% skim milk in TBST buffer (150 mM NaCl; Tris, pH 7.4; and 0.1% Tween-20) for 60 min at room temperature and then incubated with the primary antibody overnight at 4°C. Pierce ECL2 Western Blotting Substrate (Thermo Scientific) was used to visualize the blots.

### ELISA

For ELISA, culture supernatants from Infected HT1080 cells were collected at 10 hours post infection. Interferon-β was measured by ELISA kit (BD biosciences) following manufacturers’ instructions.

### Luciferase assay

HeLa cells were transfected with control UTR vector or Human STING 3’UTR Gaussia luciferase vector along with miR-24 or antimiR-24 and cells were incubated for 48 hours. The culture supernatants were collected and luciferase activity was determined by secrete pair dual luminescence kit (Genecopoeia) according to manufacturer protocol. The luciferase activity was normalized by analyzing SEAP (secretory alkaline phosphatase) activity. Ratio of luciferase activity/alkaline phosphatase activity was calculated to compare relative luciferase units.

### HSV1 infection of mice

1 nmol miRCURY LNA miRNA inhibitor was mixed with 10^4^ PFU HSV1 virus and injected intracranially into left hemisphere. Scramble negative control was mixed with virus and injected in control group. Age matched mice group injected by only HSV1 virus and PBS injected mock group were also used as control. 6 weeks old C57/BL6 mice were used for experiments bred in Cleveland clinic mice facility. Mice were monitored daily for body weight and disease symptoms. Clinical disease severity was analyzed and graded daily using the following scale: 0, no disease symptoms; 1, ruffled fur; 1.5, hunched back with mild ataxia: 2, Ataxia, balance problem and weakness of hind limb: 2.5 one leg completely paralyzed, impaired motility but still able to move around slowly; 3, severe hunching, both hind limbs paralyzed and mobility is severely compromised; 3.5 Severe distress, complete paralysis and moribund 4, dead. Day-5 post infection was chosen to compare clinical score for all groups as beyond that control group mice displayed severe morbidity.

For intraperitoneal infection experiments, 6 weeks old mice were injected with HSV1 virus intraperitoneally (10^7^ PFU) and 3 days later, 1 nmol miRCURY miRNA inhibitor or scramble control was injected intracranially and mice brain was harvested 5 days post inhibitor injection. Mice brain was homogenized using PARIS kit (Invitrogen) using manufacturer’s protocol. All animal experiments were performed in compliance with protocols approved by the Cleveland Clinic Institutional Animal Care and Use Committee.

### Quantification of western blots

Image J software was used to quantify the STING blots. The band intensity of STING was determined by software and then divided by β-actin values for normalization.

### Statistical analysis

All statistical analyses were performed using GraphPad Prism 5.02 software. The mean ± SEM of all biological replicates was used to make graphs. Statistical significance was calculated by Student’s T-test or by one way ANOVA followed by turkey’s post hoc test. Mantel-Cox test was done to compare survival curves. (P value ≤ 0.05 was considered significant, *P < 0.05; **P < 0.01, ***P < 0.001).

## Supporting information

S1 Fig miR-24 inhibits both cGAMP and HSV1 induced STING signaling. **(S1A)** Mouse RAW cells were transfected with scramble or miR-24 mimic and STING protein was analyzed by western blotting. **(S1B)** HT1080 cells were transfected with Scramble, miR-24 or antimir-24 and cGAMP was transfected 24 hours later. Interferon-β mRNA was quantified 8hrs post cGAMP transfection. **(S1C)** HT1080 cells were transfected with scramble, mir-24 or antimiR-24 and later HSV1 infection was given after 24 hours. Cells were harvested 8 hours post infection and RNA levels of IFN-β was determined. **(S1D)** HT1080 cells treated similar to (S1C) and IFIT-1 RNA level was determined. **(S1E)** Mouse RAW cells were transfected with scramble, mir-24, scramble control for antimiR or antimiR-24 and HSV1 infection was given after 24 hours. Culture supernatant and infected cells were collected after 16 hours post infection and viral titer was quantified. **(S1F)** HT1080 cells were transfected with 300 pmol 1- Scramble, 2 -miR-27b and 3- miR-24. STING expression was analyzed after 36 hrs post transfection by western blotting. **(S1G)** HT1080 cells were infected with HSV1 and cGAS expression was analyzed at various time points. (**S1B**, **S1C, S1D, S1E** Mean ± SEM, N = 3). *P<0.05, **P<0.01, ***P<0.001.
(TIF)

S2 Fig HSV1 infection does not induce expression of the miR-23a/ miR-27a/miR-24-2 locus in HT1080 cells and induces miR-24 by activating MAPK pathway. **(S2A) (S2B)** miR-23a and miR-27a expression was determined in HSV1 infected HT1080 cells. **(S2C)** HT1080 STING KD cells were infected with HSV1 and cells were harvested at various time points to determine miR-24 levels. **(S2D)** STING mRNA levels were determined in HT1080 cells 12 hours after mock (bar 1) or HSV1 (bar 2) infections; STING mRNA levels were also measured in cells 24 hours after Scramble RNA (bar 3) or miR-24 (bar 4) transfection. **(S2E)** Phospho c-Jun and phospho c-Fos levels were determined in HSV1 infected HT1080 cells. **(S2F)** Mouse NB41A3 cells were infected with HSV1 and Erk1/2 and JNK phosphorylation was analyzed by western blotting. **(S2G)** Phospho Erk1/2 activation was determined in STING KD HT1080 cells upon HSV1 infection at various time points post infection. (**S2A, S2B, S2C, S2D** Mean ± SEM, N = 3). *P<0.05, **P<0.01, ***P<0.001.
(TIF)

S3 Fig MAPKs activation induces the miR-23b/ miR-27b/miR-24-1 locus in HT1080 cells. **(S3A)** HT1080 cells were treated by PMA (10μM) and Erk1/2 and JNK activation was determined by western blotting. **(S3B)** STING protein levels were determined in PMA treated HT1080 cells by western blotting. **(S3C), (S3D)** Expression of miR-23a and miR-27a was determined in PMA treated (10μM) HT1080 cells. **(S3E), (S3F)** Expression of miR-23b and miR-27b was determined in PMA treated HT1080 cells. (**S3C, S3D, S3E, S3F** Mean±SEM, N = 3). *P<0.05, **P<0.01, ***P<0.001.
(TIF)

S4 Fig AntimiR-24 augments anti-viral response against HSV1 in mouse brain. **(S4A)** STING expression was analyzed in HSV1 infected mouse brains infected by intracranial route (day-4 and day-6 post infection). Numbers below denote densitometric quantification of STING normalized against actin. **(S4B), (S4C)** 1nmol of antimir-24 or scramble control mixed with HSV1 (10^4^ PFU) was injected intracranially into mouse brain. RNA was extracted from infected brains (day 4) and expression of Ifit-1 and Ifit-2 mRNAs was determined. (**S4B, S4C** Mean±SEM, N = 3). *P<0.05, **P<0.01, ***P<0.001.
(TIF)

S1 File The Zip file containing all graph pad prism files used for making graphs and statistical analysis. (ZIP)

S1 Table Table containing list of all primers, microRNA mimics and inhibitors used. (DOCX)
