# Supplementary figures and images for: Herpes simplex virus 1 evades cellular antiviral response by inducing microRNA-24, which attenuates STING synthesis
Source: PLoS Pathog. 2021 Sep 30;17(9):e1009950. doi: 10.1371/journal.ppat.1009950 (PMC8483329; doi:10.1371/journal.ppat.1009950)

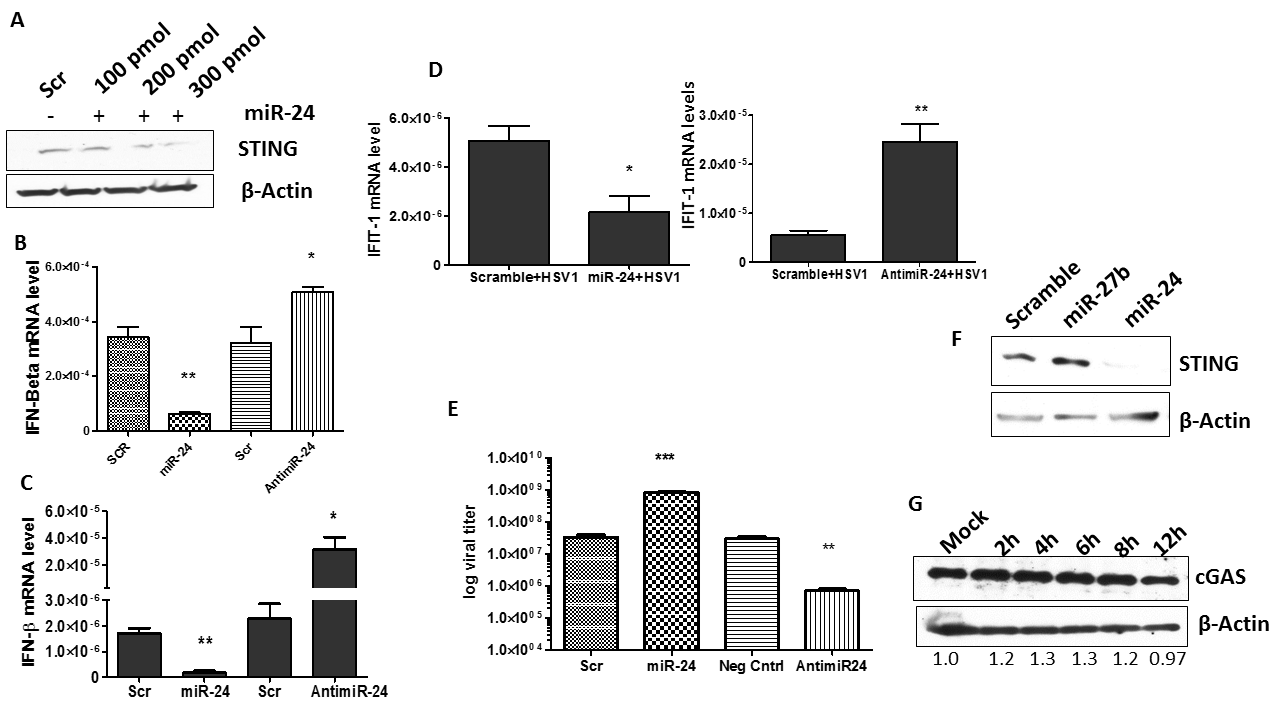

Supplement: S1 Fig — (S1A) Mouse RAW cells were transfected with scramble or miR-24 mimic and STING protein was analyzed by western blotting. (S1B) HT1080 cells were transfected with Scramble, miR-24 or antimir-24 and cGAMP was transfected 24 hours later. Interferon-β mRNA was quantified 8hrs post cGAMP transfection. (S1C) HT1080 cells were transfected with scramble, mir-24 or antimiR-24 and later HSV1 infection was given after 24 hours. Cells were harvested 8 hours post infection and RNA levels of IFN-β was determined. (S1D) HT1080 cells treated similar to (S1C) and IFIT-1 RNA level was determined. (S1E) Mouse RAW cells were transfected with scramble, mir-24, scramble control for antimiR or antimiR-24 and HSV1 infection was given after 24 hours. Culture supernatant and infected cells were collected after 16 hours post infection and viral titer was quantified. (S1F) HT1080 cells were transfected with 300 pmol 1- Scramble, 2 -miR-27b and 3- miR-24. STING expression was analyzed after 36 hrs post transfection by western blotting. (S1G) HT1080 cells were infected with HSV1 and cGAS expression was analyzed at various time points. (S1B, S1C, S1D, S1E Mean ± SEM, N = 3). *P<0.05, **P<0.01, ***P<0.001. (TIF) [file ppat.1009950.s001.tif]

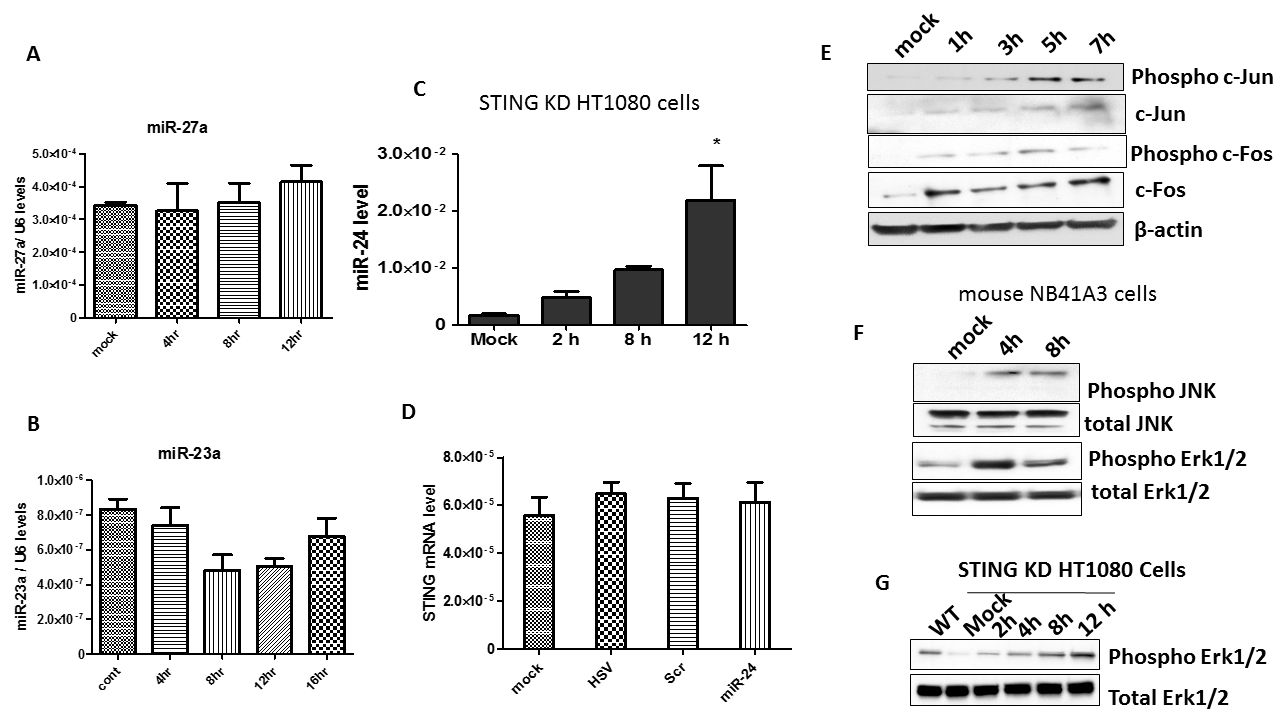

Supplement: S2 Fig — (S2A) (S2B) miR-23a and miR-27a expression was determined in HSV1 infected HT1080 cells. (S2C) HT1080 STING KD cells were infected with HSV1 and cells were harvested at various time points to determine miR-24 levels. (S2D) STING mRNA levels were determined in HT1080 cells 12 hours after mock (bar 1) or HSV1 (bar 2) infections; STING mRNA levels were also measured in cells 24 hours after Scramble RNA (bar 3) or miR-24 (bar 4) transfection. (S2E) Phospho c-Jun and phospho c-Fos levels were determined in HSV1 infected HT1080 cells. (S2F) Mouse NB41A3 cells were infected with HSV1 and Erk1/2 and JNK phosphorylation was analyzed by western blotting. (S2G) Phospho Erk1/2 activation was determined in STING KD HT1080 cells upon HSV1 infection at various time points post infection. (S2A, S2B, S2C, S2D Mean ± SEM, N = 3). *P<0.05, **P<0.01, ***P<0.001. (TIF) [file ppat.1009950.s002.tif]

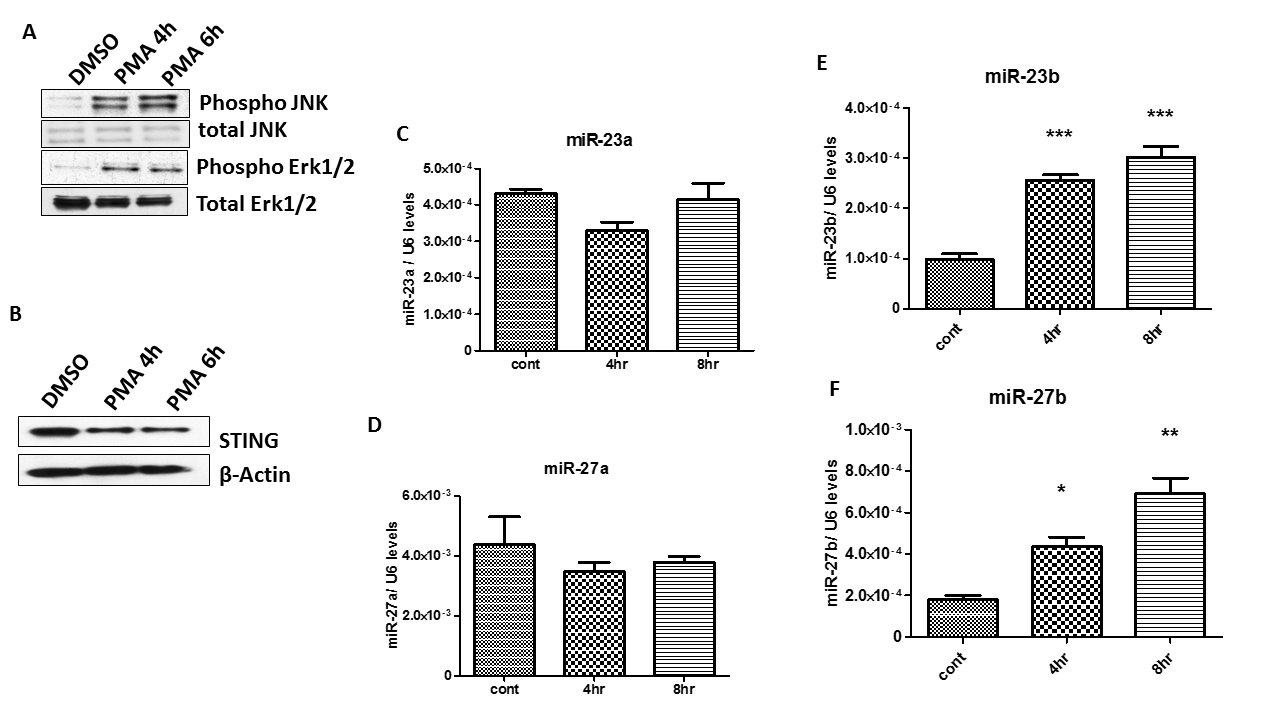

Supplement: S3 Fig — (S3A) HT1080 cells were treated by PMA (10μM) and Erk1/2 and JNK activation was determined by western blotting. (S3B) STING protein levels were determined in PMA treated HT1080 cells by western blotting. (S3C), (S3D) Expression of miR-23a and miR-27a was determined in PMA treated (10μM) HT1080 cells. (S3E), (S3F) Expression of miR-23b and miR-27b was determined in PMA treated HT1080 cells. (S3C, S3D, S3E, S3F Mean±SEM, N = 3). *P<0.05, **P<0.01, ***P<0.001. (TIF) [file ppat.1009950.s003.tif]

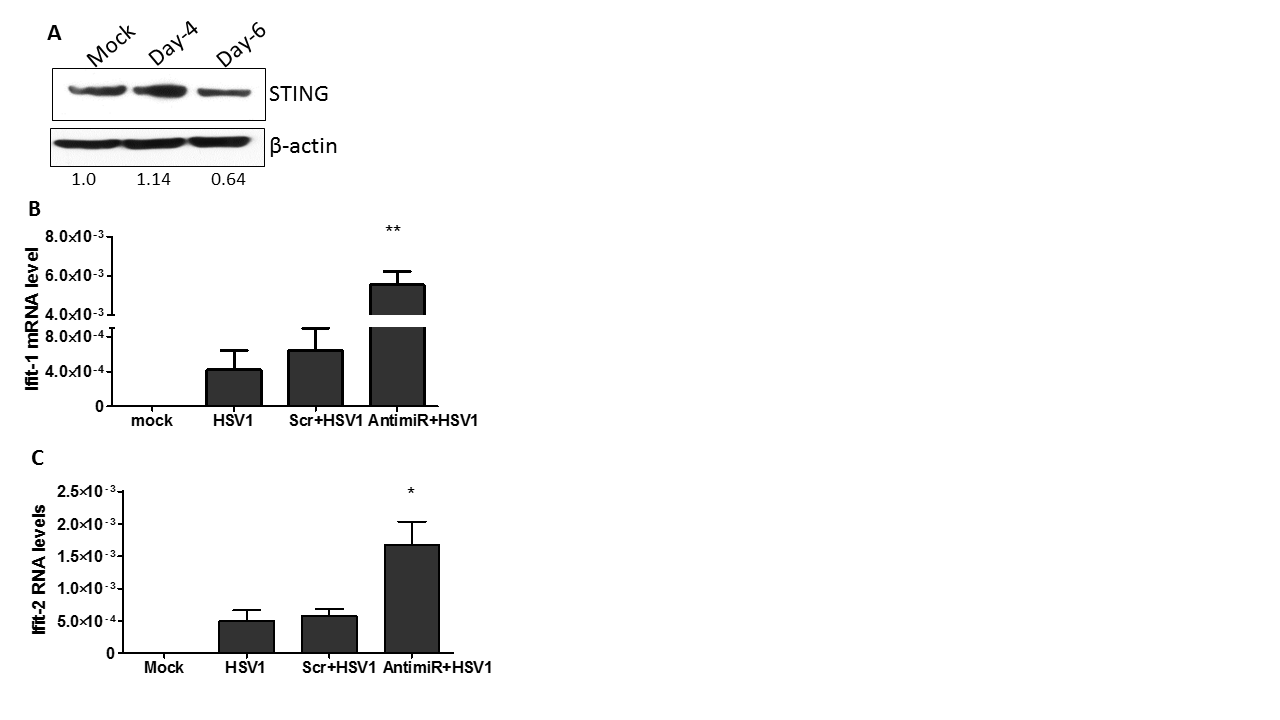

Supplement: S4 Fig — (S4A) STING expression was analyzed in HSV1 infected mouse brains infected by intracranial route (day-4 and day-6 post infection). Numbers below denote densitometric quantification of STING normalized against actin. (S4B), (S4C) 1nmol of antimir-24 or scramble control mixed with HSV1 (104 PFU) was injected intracranially into mouse brain. RNA was extracted from infected brains (day 4) and expression of Ifit-1 and Ifit-2 mRNAs was determined. (S4B, S4C Mean±SEM, N = 3). *P<0.05, **P<0.01, ***P<0.001. (TIF) [file ppat.1009950.s004.tif]
