# Supplementary material for: Herpes simplex virus 1 evades cellular antiviral response by inducing microRNA-24, which attenuates STING synthesis
Source: PLoS Pathog. 2021 Sep 30;17(9):e1009950. doi: 10.1371/journal.ppat.1009950 (PMC8483329; doi:10.1371/journal.ppat.1009950)
Supplement: S1 Table — (DOCX) [file ppat.1009950.s006.docx]

**S1 Table**- **List of primers, miRNA mimics and inhibitor**

| microRNA-24 mimic sequence | UGGCUCAGUUCAGCAGGAACAG |
| --- | --- |
| microRNA-23a mimic sequence | AUCACAUUGCCAGGGAUUUCC |
| microRNA-23b mimic sequence | AUCACAUUGCCAGGGAUUACCAC |
| microRNA-27a mimic sequence | UUCACAGUGGCUAAGUUCCGC |
| microRNA-27b mimic sequence | UUCACAGUGGCUAAGUUCUGC |
| LNA miRNA inhibitor sequence | CTGCTGAACTGAGCC |
| IFN-β primers mouse | F- CTTCTCCGTCATCTCCATAGGG  R- CACAGCCCTCTCCATCAACT |
| Ifit-1 primers mouse | F-CAGAAGCACACATTGAAGAA  R- TGTAAGTAGCCAGAGGAAGG |
| Ifit-2 primers mouse | F- CGGAAAGCAGAGGAAATCAA  R-TGAAAGTTGCCATACCGAAG |
| IFN-β primers human | F-CGCCGCATTGACCATCTA  R-GACATTAGCCAGGAGGTTCT |
| IFIT-1 primers human | F-tctcagaggagcctggctaag  R- gtcaccagaCTcctcacatttgc |
| Amplification of STING ORF vector | F- GGATCCCGCCCCTCTCCCTCCCCCC  r- TTAAGAGAAATCCGTGCGGAGAGGGAG |

| Amplification of STING 3’UTR | F- CTCTCCGCACGGATTTCTCTTAAgacccagggtcaccaggccagag  R- GAGGGAGAGGGGCGGGATCCgggtaatctgagatgtgctttaaaaaaggacc |
| --- | --- |
| Mutation of miR-24 binding site | F- tcaatcggtgtcttcaacctgtgaaatggga  R- gtaagaggggaaatgactggcccaagggga |
| AOPEP gene Exon1/2 | F- CATGCTATCAGGATATGGTAC  R- CATGTTGACATGGCAACGGG |
| STING RT PCR PRIMERS | F-CCTGAGTCTCAGAACAACTGCC  R-GGTCTTCAAGCTGCCCACAGTA |
